# Patient experiences with family medicine: a longitudinal study after the Dutch health care reforms in 2006

**DOI:** 10.1186/s12875-016-0519-7

**Published:** 2016-08-25

**Authors:** Pieter van den Hombergh, Arna van Doorn-Klomberg, Stephen Campbell, Michel Wensing, Jozé Braspenning

**Affiliations:** 1 Scientific Institute for Quality in Healthcare (IQhealth care), Radboud University Medical Center, PO Box 9101, Nijmegen, 6500 HB The Netherlands; 2 Master Physician Assistant, HAN University of Applied Sciences, PO Box 9029, Nijmegen, 6500 JK The Netherlands; 3 Centre for Primary Care, Institute of Population Health, University of Manchester, Williamson Building, Oxford Road, Manchester, M13 9PL UK; 4 Centre for Research and Action in Public Health (CeRAPH), University of Canberra, Building 22, Floor B, University Drive, Bruce, ACT 2617 Australia; 5 Department of General Practice and Health Services Research, University Hospital Heidelberg, Marsilius-Arkaden, Turm West, INF 130.3, Heidelberg, 69120 Germany

**Keywords:** Health care reform, Payment system, Patient experience, Quality of care, Practice performance, Investment in Family Medicine, Primary care

## Abstract

**Background:**

In 2006 The Dutch Health Care system changed to a market oriented system. The GP remuneration changed from ± 2/3 capitation patients and 1/3 private patients before 2006 to a mixed payment scheme. From 2006 onward every patient was insured and the GP received partly capitation, partly fees for consultations and for specific services. This change coincided with many other organisational changes in General Practice care. Our research question was if during the years after 2006 patient experiences of Dutch family practice had changed. We also wanted to explore the influence of patient and practice characteristics on patient experiences. Data on patient experiences were available from 2007 to 2012.

**Method:**

In a series of annual cross sectional patient surveys the performance of GPs and practices was measured. Patient sampling took place as a part of the Dutch accreditation program in 1657 practices involving 2966 GPs. Patients’ experiences, gender, age, health status, and number of annual consultations were documented as well as the type and location of practices. Linear regression analysis was used to examine time trends in patient experiences and the impact of patient and practice characteristics.

**Results:**

78,985 patients assessed the performance of 2966 GPs, and 45,773 patients assessed the organisation of 1657 practices. The number of patients with positive experiences increased significantly between 2007 and 2012; respectively 4.8 % for GPs (beta 0.20 and *p* < 0.0001) and 6.6 % for practices (beta 0.10, *p* < 0.004). Higher age, having no chronic illness, more frequent consultations and attending single-handed practices, predicted better patient experiences.

**Conclusions:**

In our evaluation of patient experiences with general practice care from 2007 to 2012 we found an increase of 4.8 % for GPs and 6.6 % for practices respectively. This improvement is significant. While no direct causation can be made, possible explanations may be found in the various reforms in Dutch family practice since 2006. More insight is needed into key determinants of this improvement before policymakers and care providers can attribute the improvement to these reforms.

## Background

In 2006 The Dutch Health Care system changed to a market oriented system (Table 1). The GP remuneration changed from a 2/3 capitation and 1/3 private patients before 2006 to a mixed payment scheme. From 2006 onward every patient was insured and the GP received partly capitation, partly fee for consultations and for specific services. Between 2005 and 2007 costs for GP care rose 11,2 % yearly before slowing down to 2.7 % yearly till 2012 [1]. This switch coincided with other organisational changes in general practice care, which were all meant to improve quality of care e.g. an increase in the number of nurse practitioners, incentivizing diagnostic and therapeutic activities, rewarding adherence to guidelines on availability and accessibility. and strengthening vocational training (Table 2). Changes like incentivizing care and increasing staff for chronic disease management improved clinical care in some settings, but whether these changes improved patient experience is unclear [2, 3]. Clinical care and patient experiences are distinct aspects of quality. Information on the patients’ overall evaluation of the quality of family practice care following the changes in The Netherlands was lacking [4]. The data of patient experiences using Europep [5] of a large sample of practices entering the Dutch Practice accreditation scheme each year, offered an opportunity to monitor patient experiences. The study also explored the impact of patient and practice characteristics.

**Table 1:** Changes in primary health care in The Netherlands after 2006

A market-oriented health care system was introduced in 2006 together with a new system of basic health insurance replacing the previous distinction between private and public insurance. Adult citizens are obliged to choose a health insurance, for which they pay around € 1100,- per year (with only slight differences between insurers) plus additional taxation guaranteeing basic health care coverage and free access to the GP, but excluding € 350,- co-payment for specialist care (children’s healthcare is free). It amounts to an average family spending € 11.000,- or around 23 % of its income on healthcare. Insurers got purchasing power and the government withdrew from healthcare, but set strict regulations for insurers and providers. Regulated competition between healthcare providers and between health insurers was introduced for specialist care, but General Practice was exempted from this competition. General Practice income has increased since 2006 and GPs invested in premises, staff and infrastructure, including ICT and communication equipment. Their personal income increased as well. Along with the change to market-oriented financing the total budget for GP-care rose from € 1.922 in 2006 to 2.372 million in 2010, an increase of 14 %. In 2011 all insurers invested another 10 %. Before 2006 the macro budget for GP care had been constant. Until 2006 GPs received capitation payments for their public patients (2/3), and fees per consultation for their private patients (1/3). From January 2006, GPs are being paid according to a uniform, mixed payment scheme. GPs receive a partial capitation for each patient per year plus fees per consultation for basic day care. They receive ancillary payments (mainly on a fee-for-service basis) for additional or special therapeutic and diagnostic services and for the care for chronic diseases. They are compensated on an hourly basis for care during out-of-office-hours (evening, night and weekend care). The income of self-employed Dutch GPs was ± € 96.000 in 2005 and of GPs employed by other GPs € ± 73.000.

**Table 2:** Changes in the practice organisation and in the training of GPs

- GPs started to be organized in large care groups contracting chronic care in disease management programs. From 2006 onwards the availability of a nurse practitioner for chronic disease management rose from a few percent to over 90 % (treating chronic diseases: Diabetes Mellitus, Chronic Obstructive Pulmonary Disease, Cardiovascular Disease, Mental Care), accounting for part of the rise in practice income. Between 2007 and 2012 practice nurses’ time rose from 5.5 to 11.0 h per 1000 patients per week. - Broadening the diagnostic and therapeutic scope of the practice followed the selective incentives for extra services (± € 50 per service for minor surgery, spirometry, EKG, Cyriax injection, etc.). - Primary care practices became larger with more GPs working in one center. The number of single handed practices dropped between 2006 and 2012 from 46 % to 39 %. The number of GPs rose slightly from 8612 in 2006 to 8879 in 2012 (3 %) and patients per fte GP decreased slightly. Self-reported GP-time rose from 21 to 28 h per 1000 patients per week. - In 2008, the Dutch Association of Family Medicine (LHV) accepted new guidelines on availability and accessibility. Insurers offered € 4,- for each patient when the guidelines were met. Practices should minimally be open 6 h a day, 5 days a week and address emergency calls by a medically trained person within 30 s. The GP had to visit the emergency patients within 15 min. It was incentivized but also checked by the Dutch Inspection of Health Care and subsequent failure to meet the standard was financially penalized (in practices of > 2500 patients over € 10.000, - could be missed). Only 3 practices finally did not meet the target. (personal communication L. Rijkers, LHV) - A 5 year extensive project to renew the FM-training including training the trainers was completed in 2006 with a focus on assessing and improving consultation skills. The vocational training program of GPs involved ± 1600 trainers and ± 3000 trainees. Nearly half of the GP-population thus got extra education in communication and in treating according to clinical guidelines in a new curriculum of 8 days every year.	

The Europep questionnaire has proved to be sufficiently responsive to detect changes in countries with various health policy decisions [6]. The study of Petek et al. also used Europep and compared patients with cardiovascular disease in eight European countries in 2009 with a subgroup of patients with self-defined chronic illness from their study in 1998 [7]. It showed no overall trends for the eight countries combined, but some changes in specific countries.

Allan at al. looked at the effect of Continuous Quality Improvement (CQI) on patient satisfaction using patient questionnaire data collected in a Patient Participation Program of the RACGP in Australia with a 10 year follow-up (1993–2003) [8]. They found no significant change in satisfaction but the scores showed little variation (often close to 100 % from the start). We did not find other long term studies showing measurable improvement in patient experience following organisational interventions. We therefore hypothesized that we would not find significant changes in patient experience in the years 2007-2012 in our study.

## Method

### Study design and setting

The data were collected as part of the Dutch accreditation program (NPA) between 2007 and 2012. The study focused on the patient survey that was part of the data collection preceding the Practice visit. Participating in the NPA was voluntary, yet strongly supported by the Dutch College of General Practitioners, incentivized by the insurers and becoming a future reregistration obligation for GP-trainers. The incentives stimulated hundreds of practices to enter the NPA-program each year [9]. The central outcome measures were the scores on the Dutch Europep questionnaire, which measures patient experiences with the GP- and the practice organisation. Patients (>18 years) who visited the practice were asked by the practice assistant to complete the questionnaire in the waiting room before or after the consultation, and to drop it in a sealed box warranting anonymity. The practice staff was asked to make sure that close to 30 questionnaires per GP were returned. The results of the questionnaire were used only for internal feedback for the GP and the team.

### Participants

Participants were 30 invited patients per GP entering the accreditation program.

### Measures

The Europep questionnaire has 23 items; 17 items on the GP performance and 6 items on the practice management [5]. The items of Europep are a concise reflection of what patients view as important aspects of general practice care across European countries. All items use a Likert scale (1 = poor and 5 = excellent), and a sixth option “do not know/ not applicable”. To warrant anonymity no patient characteristics were asked for except gender and age. From 2009 to 2012 the questionnaires had additional questions on percentage with chronic illness and consultation rate to allow more in depth analysis. Europep was validated in several studies and has proved to show relevant variation on all items [10]. Practice and respondents characteristics are in Tables 3 & 4.

**Table 3:** Practice characteristics compared to the national average

Practice characteristics	Study population, percentage							National Average ^b^
	2007	2008	2009	2010	2011	2012	Total	
Practice type								
Single handed	26.5 %	23.2 %	29.9 %	31.7 %	27.2 %	12.5 %	24.3 %	39.5 %
Duo or group	73.5 %	76.8 %	70.1 %	68.3 %	72.8 %	87.5 %	75.7 %	60.5 %
Urbanization degree								
- *High urbanisation* ^*d*^	45.5 %	42.5 %	43.8 %	42.3 %	44.6 %	-	44.2 %	47.7 %
- *Moderate urbanisation* ^*d*^	44.0 %	37.2 %	43.2 %	46.3 %	42.7 %	-	43.1 %	40.7 %
- *Rural* ^*d*^	10.5 %	20.3 %	13.0 %	11.4 %	12.7 %	-	12.7 %	11.6 %
Mean N of pats/ practice	4228	4882	4699	4545	4714	5171	4767	4055 ^a^
Training practice	52.2 %	42.5 %	48.4 %	69.7 %	66.5 %	53.9 %	57.5 %	33 % ^c^
Number of practices	323	323	265	237	230	279	1657	
Number of GPs	323	323	435	602	540	743	2966	

^a^ Total Dutch population divided by the total number of primary care practices

^b^ NIVEL 2010 and Dutch national Compass, 2011

^c^ Capacity committee

^d^ urbanization, high: > 1.500 addresses/km^2^, moderate: 500–1500 addresses/km^2^, rural: < 500 addresses/km^2^

**Table 4:** Characteristics of the respondents compared to the national average

Characteristics of the respondents	Study population, percentage or average (SD)							National average ^a^
	2007	2008	2009	2010	2011	2012	Total	
n	-	-	8506	17,661	15,695	15,079	56.941	
Age	-	-	51 (16)	51 (17)	52 (17)	52 (17)	52 (17)	39
Percentage women	-	-	65.5 %	64.8 %	64.5 %	64.4 %	64.6 %	50.5 %
Percentage w. chronic illness	-	-	24.1 %	23.8 %	24.6 %	24.7 %	24.4 %	31.8 %
Consultation rate	-	-	4.3 (4.1)	4.4 (4.3)	4.3 (4.0)	4.3 (4.4)	4.4 (4.3)	4.2 ^b^

^a^ NIVEL 2010 and Dutch national Compass, 2011

^b^ CBS (Dutch Central Statistical Office) in 2012

### Analysis

We assessed the scores per item by calculating the percentage of people with a rating of 4 or 5 on the 5-point Likert scale [11]. The response to the category “do not know/not applicable” was excluded. Two separate means were calculated for the items regarding GP- and practice performance.

In a linear regression model using SPSS we evaluated the trend in patient experiences over time (from 2009 to 2012; 2007 and 2008 did not have sufficient data for a linear regression). Associations between time (year) and the total scores on GP- and practice performance were explored. We also analyzed the trend for each of the individual items in separate models. In addition, we used models in which we corrected for patient age, gender, whether they self-reported chronic illness, the consultation frequency of the patients and whether the practice was a single handed practice or not.

## Results

### Study population

In total 2966 GPs in 1657 practices were included. We excluded 8.0 % of the questionnaires using three exclusion criteria; respondent age (3.8 % was below 18), number of questionnaires per practice (1.0 % had less than 10 questionnaires) and repeated measurements in the same practice (3.2 %). This resulted in 78,985 questionnaires on the performance of 2966 GPs and 45,773 questionnaires on the performance of their 1657 practices. Out of 30 questionnaires on average 27 were completed per GP and 28 per practice. The study practices were reasonably representative for Dutch general practices (Table 3). Training practices were overrepresented (around 50 % with a top of 69 % in 2010).

### General trend in patient experiences

#### GP-score

**Fig. 1:**
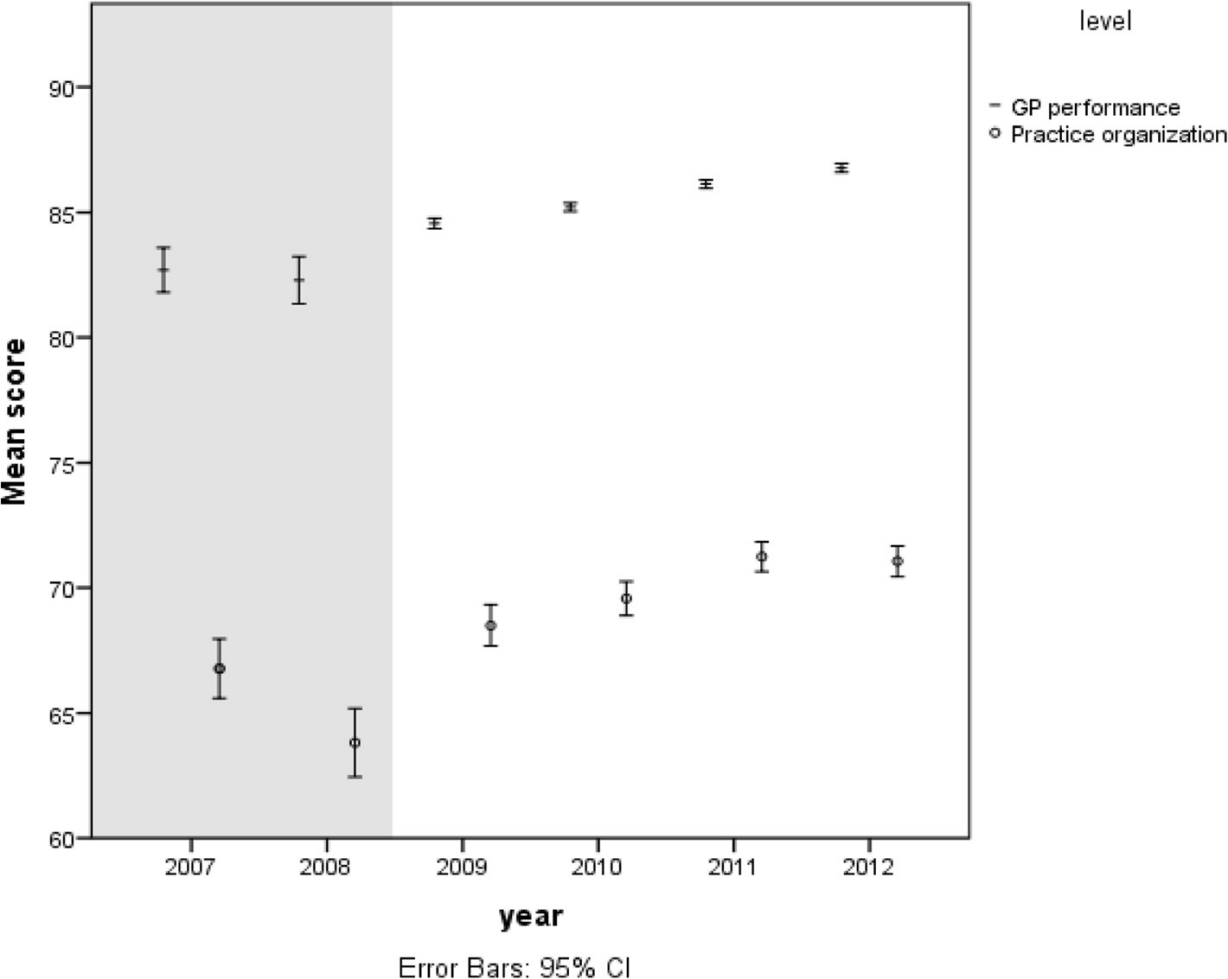
General trend in patient experience with primary care between 2007 and 2012: The grey background (2007 and 2008) are crude scores without correction for patient characteristics. The white background are scores corrected for patient characteristics (see Table 4)

Overall, more than 80 % of the patients rated the performance of their GP positively on aspects of time, empathy, listening, examining, informing, treatment and advice (Table 5). The positive trend from 82.1 % to 86.9 % (Fig. 1) over 2007–2012 is significant (Beta 0.20 and *p* < 0.001). Table 5 shows that GPs improved their care from 2009 up to 2012 regarding time for the patient, empathy, shared decision making, communication, thoroughness, patient centeredness and providing information. An analysis which corrected for differences between the cross-sectional samples regarding patient and practice characteristics confirms the positive trend between 2009 and 2012 (Beta 0,10 and *p* < 0.001).

**Table 5:** Trend of the various aspects of the Europep questionnaire from 2009 to 2012, corrected for patient age, gender, chronic illness, consultfrequency and practice type

What is your opinion of the GP and/or general practice over the last 12 months with respect to:				
	Aspect/item	Score in 2009 %	bèta	*p*
	*Composite GP score*, *n* = *2713 GPs for each item*	*84.8*	.*096*	.*0000*
1	making you feel you had time during consultations?	87.6	.085	.0001
2	interest in your personal situation?	83.3	.060	.0078
3	making it easy for you to tell him or her about your problems?	87.5	.080	.0004
4	involving you in decisions about your medical care?	84.9	.081	.0003
5	listening to you	91.4	.067	.0029
6	keeping your records and data confidential?	93.3	.078	.0005
7	quick relief of your symptoms	76.5	.069	.0020
8	helping you to feel well so that you can perform your normal daily activities?	81.0	.107	.0001
9	thoroughness	85.9	.105	.0001
10	physical examination of you?	87.6	.078	.0005
11	offering you services for preventing diseases?	79.3	.054	.0127
12	explaining the purpose of tests & treatments (screening, health checks?	87.0	.082	.0003
13	telling you what you wanted to know about your symptoms and/or illness?	87.7	.081	.0003
14	helping you deal with emotional problems related to your health status?	80.0	.058	.0098
15	helping you understand the importance of following his or her advice?	83.5	.061	.0063
16	knowing what he or she had done or told you during contacts?	81.0	.076	.0007
17	preparing you for what to expect from specialist or hospital care?	75.6	.142	.0001
	*Composite practice score*, *n* = *1527 practices for each item*	*67.8*	.*097*	.*0039*
18	the helpfulness of the staff (other than doctor) to you?	82.7	.106	.0025
19	getting an appointment to suit you?	74.6	.079	.0206
20	getting through to the practice on the telephone?	61.0	.081	.0220
21	being able to talk to the general practitioner on the telephone?	58.6	.041	.2403
22	Waiting time in the waiting room?	48.3	.062	.0670
23	Providing quick services for urgent health problems?	81.8	.114	.0010

#### Practice score

Patients rated the practice organisation slightly less positively than they rated their GP. The rating was 64.9 % in 2007 and 71.5 % in 2012 (rise of 6.6 %) (Fig. 1). This positive trend is significant (beta 0.19, *p* < 0.001) and was confirmed after correcting for differences between the cross-sectional samples in the period 2009–2012 (Beta 0.10, *p* < 0.004). Almost all items contribute to this positive trend (Table 5). The best scoring items are on “helpfulness of the staff”, “getting a suitable appointment” and “getting through to the practice on the phone”.

### Factors associated with patient experiences

Table 6 shows which factors are associated with GP performance and practice management. The year of the visit is an important explanatory factor. The trend in the period 2009–2012 in improved patient experience was confirmed after correcting for background variables.

**Table 6:** Linear regression models for both GP performance and Practice performance (2009–2012)

Variable	GP Performance		Practice Performance	
	bèta	*p*	bèta	*p*
Visitation year	.096	.0001	.097	.004
Mean age of the GP	.204	.0001	.250	.0001
%female patients	.018	.443	−.056	.098
% Patients with chronic illness	−.066	.007	−.117	.001
Mean practice consult frequency	.181	.0001	.103	.003
Single handed vs other practice	.035	.111	.269	.0001
		R2 = .078		R2 = .170

*N* = 78,985 questionnaires of 2966 GPs; *N* = 45,773 questionnaires of 1657 practices

Patient and practice factors which related to patient experiences are consistent across the year cohorts. The GP performance and practice management are rated significantly higher by older compared to younger patients and by patients with a higher frequency of consultations. Patients with a chronic disease rated the GP performance and the practice management less positively than other patients. Patients’ gender and practice urbanisation do not affect the ratings. The practice management was rated higher in single handed practices than in other types of practices, but practice type (group practice, health center) had no effect on GP performance.

## Discussion

Patient experience with Dutch general practice care changed positively in the period 2007–2012 with 4.8 % on GP personal performance and 6.6 % on practice organisation (Fig. 1). The positive change could be demonstrated for each item. The increase followed a period of profound changes in the healthcare system among which ‘investments in primary care’ and ‘the introduction of incentives in the healthcare system’. This positive trend in Dutch patient experiences is reinforced by the yearly results of the European Health Consumers Index (EHCI), that reported the Netherlands to be in the top 3 since 2007 and a first position in 2014 with a 40 points advantage over number 2 in 2014 [12].

### Changes in primary care since 2006 that could have affected patient experiences

Various bodies of research provide potential determinants for positive change in patient experiences. A Cochrane meta-analysis showed that investment in practice nurses for the treatment of chronic diseases had a positive effect on patient experiences [2]. In our practices the available time of practice nurses rose from 4.8 to 5.7 h per 1000 patients between 2009 and 2011. More time per patient proved to be associated with better patient experience in a previous study that used the same data base of practice visits [13].

A change in reimbursement for the care of chronic patients proved to be effective in the UK for clinical care [14]. But in the evaluation of the pay-for-performance (P4P) program conducted from 2003 to 2007, patients did not experience changes in quality of care for communication, nursing care, coordination, and overall satisfaction [2, 15]. Some aspects of access improved but patients reported seeing their usual physician less often and gave lower satisfaction ratings for continuity of care [3].

Patients highly value the accessibility and availability of general practice [11]. Starting in 2008 Dutch insurers incentivised service with 4 € extra capitation per patient when targets on accessibility and availability were met (Table 1). This could have helped the positive change in patient experiences on access, availability and continuity. In the UK these aspects of care did not change or worsened after P4P was introduced [15].

A positive effect on patient experience may be attributed to the change to ‘fee per consultation’ (9 € ) and ‘home visit’ (14 €) meant to compensate for the proportionate lowering of the capitation (114 € to 57 €). Although GP care was exempted from market forces and competition, the new blended system of capitation and fee for service aligning incentives more closely to professional values may have influenced patient experiences positively [14, 16].

From 2006 onward the scope of diagnostic and therapeutic procedures widened and more procedures were done in the practice also because it was financially attractive. In previous research a wider scope was associated with better medical performance in videotaped patient contacts [17].

Improvement in patient experiences may also be attributed to an extensive project to renew vocational training, which was completed in 2006. The project focused on assessing and improving trainers’ skills in giving feedback, coaching of and assessing the CANMEDS (Canadian Medical Education Directives for Specialists concerning 7 competencies: Medical Expert, Communicator, Collaborator, Manager, Health Advocate, Scholar, and Professional). Dutch GP-trainers score significantly better on quality of care and organisation including patient experiences than non-training GPs [18].

In the UK investment in family medicine training—both in GP-trainers & trainees—improved the score on the GPPS in the P4P program in the UK. GP training practice status (29 % of practices) was a significant predictor of positive GPPS responses to all questions in the ‘doctor care’ (*n* = 6) and ‘overall satisfaction’ (*n* = 2) domains but not to any of the ‘nurse care’ or ‘out-of-hours’ domain questions [19]. Doctors in GP training practices appeared to offer more patient-centered care with patients reporting more positively on attributes of doctors such as ‘listening’ or ‘care and concern’.

Extra patient time, introducing practice nurses, enhancing accessibility and availability, changing some payments in fee for services, improving consultation skills during vocational training all could have attributed to positive patient experiences. A negative effect could be expected from the increase in practice size between 2007 and 2012. Larger practices with more GPs have less positive experiences with care [6] and many patients prefer smaller practices [20, 21].

### Strengths & limitations

The data were collected within the Dutch accreditation program with a voluntary participation of practices and with maybe better practices entering first. This selection of the better practices entering first is at odds with the increase of improved patient experience. However, year after year practices who felt ready for the practice visit entered the program. This yearly mix of training and non-training practices is a selection of voluntary practices, but we had expected a negative change in patient experience in the later years, because less ambitious practices entered later in the program. We consider this a strength of this study.

Another strength is the large and representative sample of practices that could be analysed and the high numbers of new patients each year that completed the questionnaires. Selection bias due to selecting patients would very likely to have been constant over the years.

Unfortunately we could only do a linear regression analysis for the years 2009–2012, because we lacked sufficient data in 2007 & 2008.

A point of discussion was that feminisation of the profession could have contributed to the improvement. However, the role of gender was doubtful in other studies [22–24].

### Comparison with other studies

Previous studies of patient experience did show no change or mixed results after organisational change. All but one of these studies concerned P4P-studies, whereas our study included all patients who had an appointment with their doctor. Our findings that a higher age, a ‘higher frequency of consulting the GP’, ‘having no chronic illness’ and ‘a short waiting time for the consultation’ were associated with more positive patient experiences on GP performance resonate with similar analyses in previous studies [8, 24–26].

### Implications

The positive change in patient experiences with family practice cannot be related to the interventions in General Practice care. Patient experience was found to be correlated to better clinical quality in hospitals in a review by Price et al. Research indicates that better patient care experiences are associated with higher levels of adherence to recommended prevention and treatment processes, better clinical outcomes, better patient safety within hospitals, and less health care utilisation [27]. In primary care the correlation between patient experience measured with national General Practice Patient Survey (GPPS) and the national pay-for-performance scheme (QOF) was weak. The 2 domains of quality of care remain predominantly distinct [19].

Longitudinal data collection on patient experiences should span longer periods with a standardized and validated instrument such as Europep, to allow comparison over the years. Such data could enable GPs and policymakers to make better choices on practice organisation, e.g. optimal list size, being a training practice, optimal staff, etcetera [28].

## Conclusion

In our evaluation following the trend in patient experiences from 2007 to 2012 we found an increase of 4.8 % for GPs and 6.6 % for practices respectively. This is considerable given the often reported limited range for improvement in patient experience surveys. Most previous studies of patient experiences over time showed no or mixed results.

Though an attribution of the reforms to the improvement of patient experiences is impossible on the basis of this research, it is important to study the changes in Dutch Family Medicine preceding the improved patient experiences . The literature yields as possible contributors to improved patient experience: 1. The introduction of a practice nurse for chronic diseases, 2. incentivizing accessibility and availability, 3. change to a mixed capitation and fee for service payment + incentivizing additional diagnostic and therapeutic services and 4. improvement of the vocational FM-training. Policymakers and professionals could benefit from monitoring patient experiences.

## Abbreviations

CQI: Continuous Quality Improvement
EHCI: European Health Consumers Index
FM: Family Medicine
GP: General Practitioner
LHV: Dutch Association of Family Medicine
NHG: Dutch College of General Practitioners
NPA: Dutch Accreditation Program
